# Phase I/Ib Study of Tenalisib (RP6530), a Dual PI3K δ/γ Inhibitor in Patients with Relapsed/Refractory T-Cell Lymphoma

**DOI:** 10.3390/cancers12082293

**Published:** 2020-08-15

**Authors:** Auris Huen, Bradley M. Haverkos, Jasmine Zain, Ramchandren Radhakrishnan, Mary Jo Lechowicz, Sumana Devata, Neil J. Korman, Lauren Pinter-Brown, Yasuhiro Oki, Prajak J. Barde, Ajit Nair, Kasi Viswanath Routhu, Srikant Viswanadha, Swaroop Vakkalanka, Swaminathan P. Iyer

**Affiliations:** 1 Department of Dermatology, the University of Texas, MD Anderson Cancer Center, Houston, TX 77030, USA; aohuen@mdanderson.org; 2 Department of Medicine, Division of Hematology, University of Colorado, Denver, CO 80204, USA; BRADLEY.HAVERKOS@cuanschutz.edu; 3 Department of Lymphoma, City of Hope Comprehensive Cancer Center, Duarte, CA 91010, USA; jazain@coh.org; 4 Department of Lymphoma, the University of Tennessee Graduate School of Medicine, Knoxville, TN 37920, USA; RRamchandren@utmck.edu; 5 Department of Lymphoma, Emory University, Atlanta, GA 30322, USA; mlechow@emory.edu; 6 Medical College of Wisconsin. Previously with Department of Internal Medicine, University of Michigan, Ann Arbor, MI 48109, USA; sdevata@mcw.edu; 7 Department of Dermatology, Case Western Reserve University and University Hospitals Cleveland Medical Center, Cleveland, OH 44106, USA; Neil.Korman@uhhospitals.org; 8 Department of Medicine and Dermatology, Chao Family Comprehensive Cancer Center University of California, Irvine, CA 92868, USA; lpinterb@uci.edu; 9 Genentech Inc. Previously with Department of Lymphoma and Myeloma, the University of Texas M.D. Anderson Cancer Center, Houston, TX 77030, USA; okiyasuhiro@gmail.com; 10 Rhizen Pharmaceuticals S.A., CH-2300 La Chaux-de-Fonds, Switzerland; pjb@rhizen.com (P.J.B.); an@rhizen.com (A.N.); kr@rhizen.com (K.V.R.); srikantv@rhizen.com (S.V.); sv@rhizen.com (S.V.); 11 Department of Lymphoma and Myeloma, the University of Texas, MD Anderson Cancer Center, Houston, TX 77030, USA

**Keywords:** maximum tolerated dose, dose limiting toxicity, PTCL, CTCL

## Abstract

Tenalisib (RP6530), a dual phosphoinositide 3-kinase δ/γ inhibitor was evaluated in a phase I/Ib study for maximum tolerated dose (MTD), pharmacokinetics, and efficacy in patients with relapsed/refractory peripheral and cutaneous T-Cell Lymphoma (TCL). Histologically confirmed (TCL) patients, with ≥1 prior therapy received Tenalisib orally in a 28-day cycle in doses of 200 to 800 mg twice daily (800 mg in fasting and fed state) in escalation phase (*n* = 19) and 800 mg twice daily (fasting) in expansion phase (*n* = 39). The most frequently reported treatment emergent adverse events (TEAE) and related TEAE were fatigue (45%) and transaminase elevations (33%), respectively. Most frequently reported related Grade ≥3 TEAE was transaminase elevation (21%). Two dose-limiting toxicities occurred in the 800 mg fed cohort; hence, 800 mg fasting dose was deemed MTD. Tenalisib was absorbed rapidly with a median half-life of 2.28 h. Overall response rate in 35 evaluable patients was 45.7% (3 complete response (CR); 13 partial response (PR)) and median duration of response was 4.9 months. Responding tumors showed a marked downregulation of CD30, IL-31 and IL-32α. With an acceptable safety and promising clinical activity, Tenalisib can be a potential therapeutic option for relapsed/refractory TCL. Currently, a phase I/II combination study with romidepsin is ongoing.

## 1. Introduction

Peripheral T-cell lymphomas (PTCL) comprises of a heterogeneous group of lymphoid malignancies and represent approximately 12% of all non-Hodgkin lymphomas (NHLs) [1]. In the United States, PTCL not otherwise specified (PTCL-NOS) and cutaneous T-cell lymphoma (CTCL) are the most common subtypes, accounting for 15% and 27% of PTCL and CTCL cases, respectively [2,3,4,5]. The majority of PTCLs have an aggressive behavior and poor outcomes with an overall five-year survival rate of 30% [5,6,7]. CTCLs are generally indolent, with a subset having poorer outcomes. Relapsed/refractory patients with advanced-stage CTCL are usually resistant to chemotherapy and clinical response is achieved in only 30% to 40% of patients, displaying poor prognosis [8,9]. The Food and Drug Administration (FDA) approved single agents have an overall response rate (ORR) of about 30% (pralatrexate—29%, romidepsin—25%, belinostat—26%) except for brentuximab vedotin (ORR—68%) in CD30+ T-cells [10,11,12]. However, given the aggressive nature of PTCL and advanced-stage CTCL, novel therapeutic agents targeting critical pathways are needed to achieve better disease control in these patients.

Phosphatidylinositol 3-kinase (PI3K) is an important cellular signaling protein. The δ and γ isoforms are predominantly expressed in cells of hematopoietic origin. Modulation of this pathway by PI3K inhibitors can regulate several downstream events that drive the oncogenic processes such as cell proliferation, migration, and survival. Studies have also validated the PI3K-protein kinase B (AKT) pathway as a potential therapeutic target in PTCL [13,14,15,16].

Tenalisib (RP6530) is a novel, orally available, dual PI3K δ/γ inhibitor with nanomolar inhibitory potency and several fold selectivity over α and β PI3K isoforms. It has demonstrated apoptotic and anti-proliferative activity in patient-derived primary cell lines representative of B and T lymphoma cell lines [17]. It has also demonstrated efficacy in a mouse T-cell leukemia xenograft model and has shown ex-vivo activity in malignant patient derived primary CTCL cells [18].

Tenalisib was evaluated previously in a phase I study in patients with relapsed/refractory hematological malignancies and demonstrated acceptable safety and promising efficacy [19]. Based on the preliminary findings in patients with hematological malignancies, a phase I/Ib study was performed in patients with relapsed/refractory TCL.

## 2. Methods

### 2.1. Study Design

This was a two-part phase I/Ib multicenter study conducted across eight sites in the US. The first part was a phase I, open-label, 3 + 3 dose escalation, maximum tolerated dose (MTD) determination study in patients with relapsed/refractory T Cell Lymphoma. In this phase, both PTCL and CTCL patients were enrolled together across all cohorts. The second part was phase Ib, open label, dose expansion phase which was initiated after the MTD dose was confirmed. In this part, separate cohorts of PTCL and CTCL were enrolled. (ClinicalTrials.gov identifier: NCT02567656).

The study was planned to enroll up to 58 patients. The primary objective was to evaluate the safety, MTD and pharmacokinetics (PK) of Tenalisib. Secondary objectives were to evaluate the pharmacodynamic effects, the anti-tumor activity [ORR defined by complete response (CR) and partial response (PR) rates] and duration of response (DoR). Correlation of treatment outcomes with biomarkers were exploratory objectives.

The study protocol (RP6530-1401) was first approved by the Institutional Review Board (IRB) (Ethical code-2015-0130) of MD Anderson Cancer Center on 24 September 2015 and subsequently by other respective IRBs and was conducted according to the standards of Good Clinical Practice outlined in the International Council for Harmonization E6 (ICH E6) Tripartite Guideline, Declaration of Helsinki, and Code of Federal Regulation (CFR) Title 21 part 312. Written informed consent was obtained from all patients prior to enrollment in the study.

### 2.2. Eligibility Criteria

The entry criteria for patients with histologically confirmed relapsed/refractory CTCL/PTCL, included those who received one or more prior treatment lines, but not eligible for transplantation or standard/approved therapies and have radiologically measurable or skin evaluable disease.

Key inclusion criteria were age ≥18 years, life expectancy ≥12 weeks, eastern cooperative oncology group (ECOG) performance status ≤2, and adequate organ function. Adequate organ function were defined as hemoglobin ≥8 g/dL, absolute neutrophil count (ANC) ≥0.75 × 10^9^/L, platelets ≥50 × 10^9^/L, total bilirubin ≤1.5 times the upper limit of normal (ULN), alanine aminotransferase (ALT), and aspartate aminotransferase (AST) ≤2.5 × ULN if no liver involvement or 5 × ULN with liver involvement, and creatinine ≤2.0 mg/dL or calculated creatinine clearance ≥50 mL/min.

The key exclusion criteria were prior cancer therapy in the last three weeks or within five half-lives of that agent, whichever was shorter, stem cell transplantation (autologous-SCT within three months and allogeneic-SCT within 12 months), prior use of PI3K/mammalian target of rapamycin inhibitor/AKT/bruton tyrosine kinase inhibitor in the last 6 months, any investigational drug in the last four weeks, or current use of immunosuppressive therapy. In addition, patients with liver disease, uncontrolled severe infections/medical conditions, other active cancers, and pregnant and lactating women were also excluded.

### 2.3. Study Treatment

Tenalisib tablets were administered twice daily (BID) in a 28-day cycle. In the fasting cohorts, patients fasted 2 h before and 1 h after the drug administration. In the fed cohort, patients took tablets 30 min after breakfast and dinner. The starting dose of Tenalisib was 200 mg BID. The subsequent planned doses were 400 mg BID and 800 mg BID. The dosing schedule was chosen based on the short elimination half-life. The dose escalation continued until the MTD/optimal dose was identified based on dose limiting toxicities (DLT) and Cycle 1 Day (C1D1) PK data. The dose expansion phase was initiated once the MTD/optimal dose was confirmed. The planned enrollment was 20 patients in each of CTCL and PTCL cohorts for a total of 58 patients.

Treatment was continued in patients experiencing clinical benefit for two years unless there was progression of disease or toxicity which warranted discontinuation of therapy. All patients who showed CR, PR or Stable Disease (SD) and completed two years of treatment were moved to compassionate use protocol and followed [20].

### 2.4. Dose Limiting Toxicities

Protocol defined hematological or non-hematological toxicity was considered dose-limiting if it occurred during the first cycle (within the first 28 d) and was considered related to Tenalisib. Hematological DLTs was defined as Grade 4 anemia; Grade 4 neutropenia for >7 days, or Grade ≥3 febrile neutropenia with fever >38.5 °C [101 °F]; Grade 4 thrombocytopenia for >7 days, or Grade ≥3 thrombocytopenia associated with Grade >2 bleeding. Non-hematological DLTs were Grade ≥3 toxicity with exception of Grade ≥3 diarrhea or nausea that did not resolve to ≤Grade 2 within 48 h despite treatment, and ≥1.5 ULN of bilirubin or >3 ALT/AST elevation that did not resolve to ≤Grade 1 within 7 d, or treatment delay for ≥ 14 d due to unresolved toxicity.

### 2.5. Assessments

#### 2.5.1. Safety Assessment

Safety assessment included adverse event (AE) and serious adverse events (SAE) as per Common Terminology Criteria for Adverse Events (CTCAE), Version 4.03. During dose escalation, safety assessments were performed at weekly intervals during Cycle 1 and Cycle 2 and at two weekly intervals during Cycle 3 and 4. After completion of Cycle 4, safety assessments were performed on Day 1 of each subsequent cycle. In dose expansion phase, all safety assessments were performed on Day 1 of each cycle.

#### 2.5.2. Pharmacokinetic (PK) Assessments

Complete PK assessments were performed during dose escalation on Day 1 of Cycle 1 and 2 and pre-dose at other time points (pre-dose on Day 8 and Day 15 of Cycle 1 and 2, Day 1 and Day 15 of Cycle 3 and 4 and pre-dose on Cycle 5 and beyond). In the dose expansion phase, samples were collected pre-dose on Day 1 from Cycle 1 to Cycle 8. The PK parameters included maximum plasma concentration (C_max_), time to maximum plasma concentration (T_max_), area under the plasma concentration-time curve from time of administration to the end of the dosing interval (AUC_(0-*τ*)_), apparent oral drug clearance during a dosing interval (CL_ss_/F), apparent volume of distribution during a dosing interval (Vz/F) and elimination half-life (t_1/2_) were evaluated.

#### 2.5.3. Efficacy Assessments

Efficacy assessments included radiological, bone marrow biopsy/aspiration and skin assessments. In some cases, bone marrow disease alone was used for disease assessment. In these cases, response was reported as CR or non-CR.

In PTCL patients, radiological assessment was performed at screening, Day 1 of Cycle 3 and 5 and approximately 12 weeks thereafter. Assessments of skin lesions in PTCL patients were performed, if applicable. Bone marrow biopsy/aspirate was performed in patients without measurable but assessable disease at screening and on Day 1 of Cycle 3 and 5, then also approximately 12 weeks thereafter to confirm a CR.

In CTCL patients, assessment of skin lesions was performed at screening, Day 1 of Cycle 3 and 5, approximately 12 weeks thereafter and at the end of treatment (EOT). Skin photographs were obtained for half body global and up to 5 selected representative index lesions at baseline, at Day 1 of Cycle 3, EOT, at PR/CR/progressive disease (PD) and as required as per the discretion of the Investigator. In case of nodal involvement, radiological assessment was performed at screening, Day 1 of Cycle 3 and 5 and approximately 12 weeks thereafter. Skin biopsy was performed from the most indurated area at screening, on C3D1 and to confirm a CR. Patients with predominantly blood involvement had a bone marrow biopsy at screening and as indicated to confirm a CR.

The efficacy parameters included ORR and DoR. Revised Response Criteria for Malignant Lymphoma (Cheson 2007) was used for evaluation of PTCL [21]. International Society for Cutaneous Lymphomas Response Criteria for Mycosis Fungoides and Sezary Syndrome (SS) and Cutaneous Lymphoma Task Force of the European Organization for Research and Treatment of Cancer criteria were used for evaluation of CTCL patients [22]. For skin scoring, the modified Severity Weighted Assessment Tool was used. For local index lesion skin scoring, the Composite Assessment of Index Lesion Severity was used.

#### 2.5.4. Pharmacodynamic Assessment

Biomarker phosphorylated-AKT (pAKT) was to be estimated in five Sezary syndrome patients from single center (MD Anderson Cancer Centre, Houston, TX, USA) in dose escalation phase. Samples were to be collected pre-dose and after 1 h on Day 1 of Cycle 1, pre-dose on Day 8 and 22 of Cycle 1, Day 1 of Cycle 2 and 3 and at EOT.

#### 2.5.5. Correlative Biomarker Analysis

Assessment of CD30 and soluble IL-2 receptor (sIL-2R) for PTCL and cutaneous T-cell attracting chemokine (CTACK), IL-31 and IL-32α for CTCL were performed in plasma at screening, pre-dose and after 1 h on Day 1 of Cycle 3, at EOT and/or to confirm a response.

### 2.6. Statistical Analysis and Sample Size

No formal sample size calculation was performed for this dose escalation followed by dose expansion study. The safety population included all patients who received at least one dose of study medication. PK population included all patients of the safety population who provided at least one blood draw. Efficacy population included all patients of the safety population who provided at least one post-baseline disease assessment.

All statistical analyses were performed using SAS 9.2 (SAS Institute (India) Pvt. Ltd., Mumbai, Maharashtra, India). Descriptive statistics were provided in the summary tables as per the dose cohort. Quantitative variables were summarized by using N, arithmetic means, standard deviation, median and range. Categorical variables were summarized by using frequency distributions and percentages, and 95% confidence intervals when applicable.

## 3. Results

### 3.1. Patient Disposition and Baseline Characteristics

**Figure 1:**
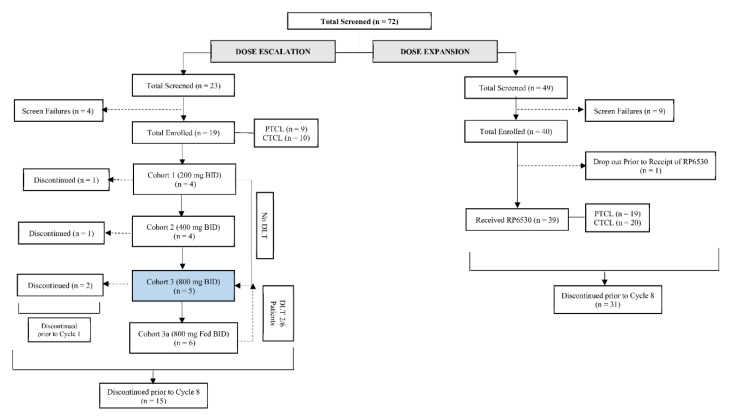
Disposition of subjects.

From October 2015 to December 2018, a total of 59 patients were enrolled across the dose escalation and expansion cohorts of which 58 patients (28 (48.3%) PTCL and 30 (51.7%) CTCL) received at least one dose of Tenalisib. Nineteen patients (9 PTCL, 10 CTCL) were enrolled in 4 cohorts of dose escalation phase [Cohort 1: 200 mg, *n* = 4; Cohort 2: 400 mg BID *n* = 4; Cohort 3: 800 mg BID (fasting) *n* = 5; and Cohort 4: 800 mg BID (fed), *n* = 6] and thirty-nine patients (19 PTCL and 20 CTCL) in expansion phase (800 mg BID fasting) in a 28-day cycle. In dose escalation phase, patients in cohort 1 to 3 received Tenalisib in a fasting state and Cohort 4 received Tenalisib in fed state (Figure 1).

The median age of the patients was 67 years. Males were 52% and females 48%. Stage IV was the most common stage in both PTCL (18 patients) and CTCL (12 patients). All patients received one or more prior cancer therapies with 81% patients having received ≥3 prior therapies. ECOG performance status was 0, 1, and 2 in 41%, 53% and 5% patients, respectively. The disease status was refractory to the last prior therapy in 38 (66%) patients whereas 20 (35%) patients had relapsed after receiving last prior therapy. The demographic and baseline characteristics for all patients who received Tenalisib are given in Table 1.

### 3.2. Safety

The median duration of exposure in the dose escalation phase was 99 d (range 0.8 to 641 d) and in the dose expansion phase was 67 d (range 13 to 644 d). Safety assessment of 58 patients receiving at least one dose of Tenalisib demonstrated an acceptable safety profile. In the dose escalation phase, no DLTs were noted in fasting Cohorts 1, 2, and 3. Two DLTs (Grade 3 ALT increased and Grade 3 erythematous rash) were observed in Cohort 4 (fed) and hence this dose was considered exceeding the MTD. The dose of 800 mg BID (fasting) was considered as MTD and dose expansion phase was initiated with this dose.

Twelve patients (four in dose escalation phase and eight in dose expansion phase) completed at least 8 cycles while others were discontinued prior to Cycle 8. Four patients of dose expansion phase were rolled over to the compassionate medication study. The most common reason for discontinuation was disease progression (61%) followed by adverse events (9%), consent withdrawal (9%), investigator’s discretion (9%), intercurrent illness (3%) and non-compliance to trial requirements (2%).

Overall, 57 (98%) patients had at least one treatment emergent adverse event (TEAE) of which 45 (78%) patients had at least one related TEAE [17 (90%) in dose escalation phase and 28 (72%) in dose expansion phase]. In both phases the most frequently reported TEAE was fatigue reported in 26 (45%) patients. This was followed by AST increase in 21 patients (36%), ALT increase in 20 patients (35%) and diarrhea in 19 patients (33%) (Table 2). Among the related TEAEs, 7 (37%) patients in dose escalation phase and 17 (41%) patients in dose expansion phase reported Grade 3 TEAEs, while three (5.2%) patients in dose expansion phase reported Grade 4 TEAEs. All other related TEAEs were of Grade 1/2 in intensity (Table S1). Transaminase elevations (21%) were the most frequently reported related Grade ≥3 TEAE in both phases and most of the events were reported within Cycle 2. Anemia (8.6%), neutropenia (6.9%) and hyponatremia (6.9%) were the other Grade 3 TEAEs (Table 3). Grade 4 related TEAEs included two events of ALT increase and one event of sepsis. Most of these AEs were reversed by withholding the dose of Tenalisib and with supportive management. Additionally, steroids were used in nine patients to manage elevated ALT/AST. None of the related TEAEs led to a fatal outcome.

Thirty-seven SAEs were reported in 22 (38%) patients in the study. Of these, six SAEs (pyrexia, International Normalization Ratio increase, sepsis syndrome, diplopia secondary to neuropathy, hypersensitivity, and skin infection) in dose expansion phase was assessed to be related to the Tenalisib. The most frequently reported SAEs were related to infections in nine (16%) patients followed by gastrointestinal disorders in five (9%) patients.

A total of seven (12%) deaths were reported in the study; however, none of them were related to Tenalisib. The various causes of death included hyponatremia, lung infection and sepsis in patients who had disease progression. One death was attributed to auto-immune hemolytic anemia. The remaining three patients expired in hospice/home after being discontinued from the study due to disease progression. Two patients who experienced DLTs; were continued in the study at reduced dose (400 mg BID).

Eight TEAEs led to discontinuation of eight (14%) patients. Five TEAEs (two events of ALT increased, rash, diplopia secondary to neuropathy, and sepsis) were related to Tenalisib and three TEAEs (event of myelodysplastic syndrome, autoimmune hemolytic anemia, and increased AST) were not related to Tenalisib.

### 3.3. Pharmacokinetics

Tenalisib was rapidly absorbed within 2 h. C_max_ and AUC_(0-*τ*)_ increased proportionally with dose on Cycle 1 Day 1. In the fasting cohorts, a 1.7-fold and 1.8-fold increase were observed in C_max_ between 200 mg and 400 mg BID and between 400 mg and 800 mg BID treatment cohorts on Cycle 1 Day 1 respectively. Corresponding increases on Cycle 2 Day 1 were 1.8-fold and 1.1-fold. A 1.8-fold and 2.6-fold increase was observed in AUC_(0-*τ*)_ between 200mg and 400 mg BID treatment cohorts, and 400 mg and 800 mg BID treatment cohorts on Cycle 1 Day 1 respectively. Exposures generally trended higher in 800 mg BID fasting cohort compared to 800 mg BID fed cohort, this difference was however not significant (i.e., >20%) to show a food effect.

There was no significant accumulation between Cycle 1 Day 1 and Cycle 2 Day 1. Apparent CLss/F were similar for all cohorts and ranged from 0.05–0.07 L/h with a short median t_1/2_ of 2.28 h (range 1.6 to 2.8 h). Tenalisib was not highly distributed in tissues with Vz/F similar for all cohorts even though the fed state trended a bit higher on distribution (Table 4).

### 3.4. Pharmacodynamic Assessment

As there was no Sezary syndrome patient in the dose escalation cohort at MD Anderson Cancer Center, pAKT analysis was not done.

### 3.5. Efficacy

Total 16 patients (7 in dose escalation and 9 in dose expansion) out of 35 evaluable patients in the study responded to Tenalisib. ORR was 45.7% (95% CI: 28.8–63.4) among responders. CR was reported in three (9%) patients while PR was noted in 13 (37%) patients. A total of 11 (31%) patients experienced SD while 8 (22.9%) experienced PD. Most of the patients responded at Cycle 3 Day 1.

**Figure 2:**
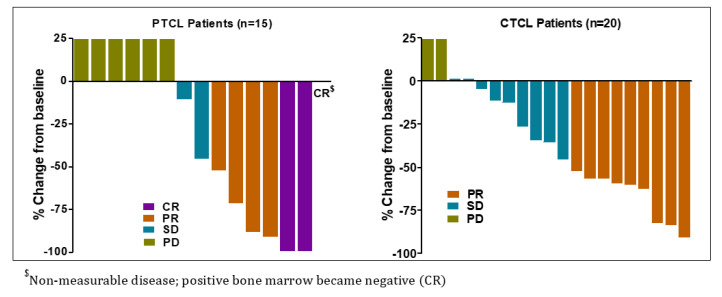
Best response(s) to Tenalisib in PTCL and CTCL evaluable patients.

The response among PTCL and CTCL patients were similar [PTCL 46.7% (95% CI: 21.3, 73.4), CTCL 45% (95% CI: 23.1, 68.5)]. Three (20%) PTCL patients achieved CR and 4 (26.7%) achieved PR while 9 CTCL patients achieved PR. The overall median DoR was 4.9 months (95% CI: 4.3, 12.0) with 6.5 months (95% CI: 2.9, 14.9) in PTCL and 3.8 months (95% CI: 2.3, 12.8) in CTCL patients (Table 5 and Figure 2). In three PTCL patients who achieved CR, the median DoR was 13.3 months (range 7.5–18.6).

### 3.6. Biomarker Analysis

**Figure 3:**
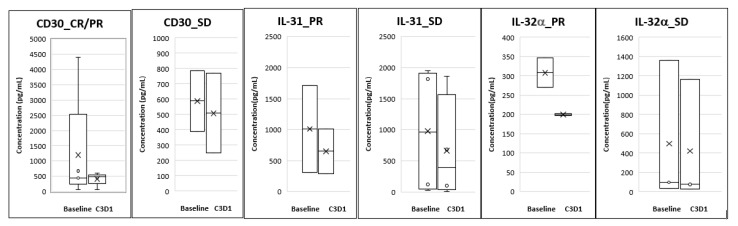
Correlative biomarkers of patients treated with Tenalisib. The data is presented as mean (denoted by X) and Standard Error of Mean.

Analysis of plasma biomarkers from PTCL patients showed a decrease in CD30 levels from baseline to Cycle 3 Day 1 and this correlated with response seen in these patients. For the CTCL group, reductions were seen in plasma IL-31 and IL-32α levels at Cycle 3 Day 1 compared to baseline and this reduction correlated with the responses seen (Figure 3).

## 4. Discussion

In relapsed/refractory T-cell lymphomas, blockade of PI3K δ/γ isoforms at clinically achievable concentrations could potentially inhibit T-cell activation/proliferation via inhibiting the δ isoform and alteration of tumor microenvironment by inhibiting the γ isoform. Therefore, dual targeting of PI3K δ/γ is a promising strategy in lymphomas. Tenalisib is a highly specific and orally available dual PI3K δ/γ inhibitor with nanomolar inhibitory potency and several fold selectivity over α and β isoforms.

Tenalisib has been previously evaluated in a European study in relapsed/refractory patients with hematological malignancies and showed a favorable safety profile with low incidence of adverse events and minimal discontinuation due to TEAEs [19]. The safety profile of Tenalisib in the current study was different from that in the European study, which did not show adverse events, such as transaminitis and skin rash. This could be attributed to the different patient populations enrolled in these two studies. In addition, the European study largely enrolled patients with B-cell malignancies that could potentially account for the differences. An integrated safety analysis performed across the two studies [23] showed that the overall incidence of the TEAEs were lower than those reported with other PI3K inhibitors that are in development or have been FDA approved [15,24,25]. Cumulative safety data revealed that overall incidence of immune mediated toxicities appears lower with Tenalisib (transaminitis 10%; no events of colitis and pneumonitis) as compared to other PI3K inhibitors (duvelisib–transaminitis 10–40%, colitis 11–18%, pneumonitis 5–20%, idelalisib-transaminitis 16–18%, colitis 14–20%, pneumonitis 4%) [15,24,25].

In the current study, transaminase (ALT/AST) elevations usually occurred in the first eight weeks of therapy. Most of these events were reversed by dose interruptions/dose reductions and supportive therapy (e.g., steroid treatment). Unlike other PI3K inhibitors, late onset toxicities like colitis, pneumonitis, and opportunistic infections were not seen with Tenalisib even in those patients treated for more than six months, although numbers were small (29%) [23]. Five patients were on therapy for more than 18 months and did not report any late onset immune toxicities. Though the data is limited at this juncture, there may be a different late onset safety profile emerging for Tenalisib.

In this study, the overall incidence of AEs was similar in PTCL and CTCL patient groups. However, the frequency and intensity of related AEs were higher in CTCL than in PTCL patients. A total of 27 (90%) CTCL patients experienced 208 related AEs whereas 18 (64%) PTCL patients experienced 51 related AE. Similarly, 12 (40%) CTCL patients experienced 25 related ≥Grade 3 AEs and 9 (32%) PTCL patients experienced 12 related ≥Grade 3 AEs. The reason for this numerically higher incidence of related AEs in CTCL patient is not clear. Further studies may be needed to understand the difference in these 2 subtypes of T-cell lymphomas.

Tenalisib was absorbed rapidly attaining peak concentrations within two hours of administration. Steady-state pharmacokinetics revealed no accumulation. A food effect study was conducted in healthy volunteers during the course of dose escalation [26]. Data in healthy volunteers indicated that the presence of food (high fat meal) increased the overall exposure to Tenalisib though there was no significant effect on C_max_ [27]. Based on this data, an additional fed cohort at 800 mg BID dose was added to determine food effect. Contrary to the healthy volunteer study, there was no meaningful change (<20%) in the C_max_ and AUC’s in the 800 mg fed and fasting cohorts, ruling out any food effect on Tenalisib in patients. In addition, the DLTs seen in the 800 mg BID fed cohort did not correlate with pharmacokinetics as it was similar in the fasting and the fed state in patients. Given the lack of pharmacokinetic benefit as well as the presence of two DLTs in 800 mg BID fed cohort, we concluded that 800 mg BID fasting should be pursued further for expansion cohort and for all subsequent development programs of Tenalisib.

Tenalisib showed promising anti-tumor activity in relapsed/refractory T cell lymphoma patients. The response rates were similar in CTCL and PTCL patients. Although this is the first study of Tenalisib in a T-cell population, the efficacy data seems to be similar to that of other targeted and non-targeted agents like, romidepsin, pralatrexate and belinostat in patients with relapsed/refractory PTCL/CTCL [10,12,28,29]. When compared to Duvelisib, the only PI3Kδ/γ inhibitor which has been evaluated in T cell lymphoma, Tenalisib showed comparable response rates and duration of response. Although the incidence of AEs like neutropenia and transaminitis were lower with Tenalisib, only a head to head study will enable comparisons [15,16].

Multiple studies have shown that relative decrease of sIL-2R levels correlates with time to complete response and is useful for early predictor of response in PTCL. High sIL-2R level predicts a poor prognosis in PTCL [30,31]. High expression of CD30 correlates with poor survival in patients with PTCL [32]. Similarly, CTACK level in skin and sera after therapy correlates with the risk of recurrence [33,34]. Studies have shown that IL-31 is produced by the malignant T-cells in CTCL patients and serum levels directly correlate with the degree of pruritus in leukemic CTCL patients. IL-32 levels positively correlate with disease activity in CTCL patients [35,36].

We evaluated the effect of Tenalisib on the above biomarkers which correlate with disease pathology and symptoms. There were correlative trends of reduction in CD30, IL-31, and IL-32α levels with responses. However, there were limitations in the analysis of the data since we could not correlate disease progression to these markers. This was due to unavailability of paired blood samples for patients whose disease progressed before they reached Cycle 3 Day1.

## 5. Conclusions

The safety and efficacy data support the development of Tenalisib as monotherapy or in combination with existing or novel targeted therapies in patients with hematological malignancies. On-going data from studies of Tenalisib as monotherapy in indolent NHL (NCT03711578) [37] and in combination with romidepsin in T-cell lymphomas (NCT03770000) [38] indicate that Tenalisib is well tolerated. With a favorable safety profile and promising clinical activity, Tenalisib holds promise as an emerging potential therapeutic option for patients with relapsed/refractory TCL.

## Figures and Tables

**Table 1:** Demography and baseline characteristics.

Characteristic	Dose Escalation		Dose Expansion		Total
	CTCL	PTCL	CTCL	PTCL	
Sex/Gender, *N* (%)					
Male	6 (60.0%)	5 (55.6%)	7 (35.0%)	12 (63.2%)	30 (51.7%)
Female	4 (40.0%)	4 (44.4%)	13 (65.0%)	7 (36.8%)	28 (48.3%)
Race, *N* (%)					
White/Caucasian	10 (100.0%)	9 (100.0%)	16 (80.0%)	14 (73.7%)	49 (84.5%)
Asian	0	0	0	1 (5.3%)	1 (1.7%)
Afro-American	0	0	4 (20.0%)	4 (21.1%)	8 (13.8%)
Age (years)					
Mean	69.4	57.6	63.4	68.9	65.4
Median	69.4	55.9	67.	63.9	67.1
Range	60.7–76.6	40.9–73.2	39.5–84.5	45.7–89.5	39.5–89.5
Diagnosis, *N* (%)					
PTCL	0	9 (100.0%)	0	19 (100.0%)	28 (48.3%)
CTCL	10 (100.0%)	0	20 (100.0%)	0	30 (51.7%)
ECOG Performance Status Score, *N* (%)					
0	10 (100.0%)	2 (22.2%)	7 (35.0%)	5 (26.3%)	24 (41.4%)
1	0	6 (66.7%)	12 (60.0%)	13 (68.4%)	31 (53.4%)
2	0	1 (11.1%)	1 (5.0%)	1 (5.3%)	3 (5.2%)
Disease Status, *N* (%)					
Relapse after last treatment	5 (50.0%)	3 (33.3%)	3 (15.0%)	9 (47.4%)	20 (34.5%)
Refractory to last treatment	5 (50.0%)	6 (66.7%)	17 (85.0%)	10 (52.6%)	38 (65.5%)
Stage of the Disease, *N* (%)					
Stage I	3 (30.0%)	0	7 (35.0%)	1 (5.3%)	11 (18.9%)
Stage II	1 (10.0%)	1 (11.1%)	4 (20.0%)	1 (5.3%)	7 (12.1%)
Stage III	0	3 (33.3%)	3 (15.0%)	4 (21.1%)	10 (17.2%)
Stage IV	6 (60.0%)	5 (55.6%)	6 (30.0%)	13 (68.4%)	30 (51.7%)
Prior therapies, *N* (%)					
Therapy ≥ 3	9 (90.0%)	8 (88.9%)	18 (90.0%)	12 (63.2%)	47 (81.0%)
Therapy ≥ 5	8 (80.0%)	4 (44.4%)	13 (65.0%)	2 (10.5%)	27 (46.6%)

*N* = Number of patients; PTCL = Peripheral T-cell lymphoma; CTCL = Cutaneous T-cell lymphoma; ECOG = Eastern cooperative oncology group.

**Table 2:** Incidence of adverse event (AE) (AE ≥ 10%) reported in the study (relationship-all).

System Organ Class (SOC) and Preferred Term (PT)	Dose Escalation	Dose Expansion	Total
	(*N* = 19)	(*N* = 39)	(*N* = 58)
	*n* (%), E	*n* (%), E	*n* (%), E
Gastrointestinal disorders			
Diarrhea	5 (26.3%), 6	14 (35.9%), 19	19 (32.8%), 25
Nausea	6 (31.6%), 7	9 (23.1%), 9	15 (25.9%), 16
Constipation	4 (21.1%), 5	5 (12.8%), 5	9 (15.5%), 10
Vomiting	4 (21.1%), 5	5 (12.8%), 6	9 (15.5%), 11
Abdominal pain	3 (15.8%), 3	5 (12.8%), 6	8 (13.8%), 9
General disorders and administration site conditions			
Fatigue	8 (42.1%), 8	18 (46.2%), 19	26 (44.8%), 27
Pyrexia	4 (21.1%), 4	9 (23.1%), 17	13 (22.4%), 21
Metabolism and nutrition disorders			
Decreased appetite	5 (26.3%), 5	7 (17.9%), 8	12 (20.7%), 13
Dehydration	3 (15.8%), 5	10 (25.6%), 13	13 (22.4%), 18
Hyponatremia	4 (21.1%), 4	4 (10.3%), 5	8 (13.8%), 9
Hypokalemia	4 (21.1%), 5	3 (7.7%), 5	7 (12.1%), 10
Investigations			
Aspartate aminotransferase increased	7 (36.8%), 16	14 (35.9%), 21	21 (36.2%), 37
Alanine aminotransferase increased	6 (31.6%), 13	14 (35.9%), 20	20 (34.5%), 33
Gamma-glutamyl transferase increased	2 (10.5%), 2	6 (15.4%), 9	8 (13.8%), 11
Blood creatinine increased	1 (5.3%), 1	6 (15.4%), 6	7 (12.1%), 7
Blood thyroid stimulating hormone increased	2 (10.5%), 2	4 (10.3%), 5	6 (10.3%), 7
Nervous system disorders			
Dizziness	3 (15.8%), 3	7 (17.9%), 9	10 (17.2%), 12
Headache	3 (15.8%), 4	7 (17.9%), 9	10 (17.2%), 13
Skin and subcutaneous tissue disorders			
Pruritus	4 (21.1%), 5	5 (12.8%), 5	9 (15.5%), 10
Dry skin	2 (10.5%), 2	5 (12.8%), 6	7 (12.1%), 8
Rash	3 (15.8%), 4	3 (7.7%), 6	6 (10.3%), 10
Respiratory, thoracic and mediastinal disorders			
Tachycardia	5 (26.3%), 6	3 (7.7%), 3	8 (13.8%), 9
Dyspnea	5 (26.3%), 5	2 (5.1%), 2	7 (12.1%), 7
Cough	6 (31.6%), 7	7 (17.9%), 9	13 (22.4%), 16
Musculoskeletal and connective tissue disorders			
Muscle spasms	5 (26.3%), 6	4 (10.3%), 4	9 (15.5%), 10
Blood and lymphatic system disorders			
Anemia	3 (15.8%), 6	6 (15.4%), 10	9 (15.5%), 16
Psychiatric disorders	4 (21.1%), 6	7 (17.9%), 12	11 (19.0%), 18
Insomnia	3 (15.8%), 3	3 (7.7%), 4	6 (10.3%), 7

*n* = Number of patients with at least one event; E = Count of events.

**Table 3:** Summary of Grade 3/4 AE (AE ≥ 5%) reported in the study (Relationship-all).

Significant TEAEs	Dose Expansion	Dose Escalation	Total
	(*N* = 19)	(*N* = 39)	(*N* = 58)
	*n* (%), E	*n* (%), E	*n* (%), E
Alanine aminotransferase increased	5 (26.3%), 5	6 (15.4%), 7	11 (19.0%), 12
Aspartate aminotransferase increased	4 (21.1%), 4	7 (17.9%), 8	11 (19.0%), 12
Anemia	2 (10.5%), 2	3 (7.7%), 6	5 (8.6%), 8
Neutropenia	2 (10.5%), 2	2 (5.1%), 2	4 (6.9%), 4
Hyponatremia	2 (10.5%), 2	2 (5.1%), 2	4 (6.9%), 4
Thrombocytopenia	1 (5.3%), 1	2 (5.1%), 2	3 (5.2%), 3
Fatigue	1 (5.3%), 1	2 (5.1%), 3	3 (5.2%), 4

*n* = Number of patients with at least one event; E = Count of events. TEAEs = Treatment emergent adverse events.

**Table 4:** Pharmacokinetics of Tenalisib.

Parameter	200 mg BID		400 mg BID		800 mg BID		800 mg BID (fed)	
	C1D1	C2D1	C1D1	C2D1	C1D1	C2D1	C1D1	C2D1
C_max_ * (ng/mL)	1297.3	1651.4	2196.7	2897.7	3995.7	3231.5	2668.0	4165.6
AUC_0–∞_ * (h * ng/mL)	3538.3	4018.0	6530.9	5603.9	17,363.7	17,159.4	14,538.5	14,233.8
AUC_(0-*τ*)_ * (h * ng/mL)	3503.0	3970.8	6415.5	5526.8	16,424.2	16,200.3	13,591.2	13,555.3
T_max_ # (h)	1.0	0.5	0.8	0.5	1.1	2.1	2.1	1.1
t_1/2_ * (h)	1.6	1.8	1.9	2.0	2.6	2.6	2.6	2.8
λ_z_ (h^−1^)	0.4	0.4	0.4	0.4	0.3	0.3	0.3	0.3

C_max_ = Maximum plasma concentration; AUC_0-∞_ = Area under the curve to infinity; AUC_(0-*τ*)_ = Area under the plasma concentration-time curve from time of administration to the end of the dosing interval; T_max_ = Time taken to reach maximum concentration; t_1/2_ = Elimination half-life. λ_z_ = Apparent terminal elimination rate constant; BID = Twice a day; C = Cycle; D = Day. * Geometric mean; # Median.

**Table 5:** Summary of overall response rate and duration of response.

Population	Statistic	Dose Escalation					Dose Expansion	Cumulative
		200 mg	400 mg	800 mg Fasting	800 mg Fed	Total	800 mg Fasting	Total
All	*N*	*N* = 3	*N* = 2	*N* = 3	*N* = 5	*N* = 13	*N* = 22	*N* = 35
	ORR (95% CI) (%)	33.3 (0.8, 90.6)	100.0 (15.8, 100.0)	66.7 (9.4, 99.2)	40.0 (5.3, 85.3)	53.8 (25.1, 80.8)	40.9 (20.7, 63.6)	45.7 (28.8, 63.4)
	Median DoR (95% CI) (months)	4.9 (-,-)	2.4 (0.97, 3.8)	10.9 (2.3, 19.5)	3.3 (1.7, 4.9)	3.8 (0.7, 10.2)	7.5 (4.7, 15.9)	4.9 (4.3, 12.0)
CTCL	*N*	*N* = 1	*N* = 1	*N* = 2	*N* = 4	*N* = 8	*N* = 12	*N* = 20
	ORR (95% CI) (%)	0.0 (0.0, 97.5)	100.0 (2.5, 100.0)	50.0 (1.3, 98.7)	50.0 (6.8, 93.2)	50.0 (15.7, 84.3)	41.7 (15.2, 72.3)	45.0 (23.1, 68.5)
	Median DoR (95% CI) (months)	--	3.8 (-,-)	2.3 (-,-)	3.3 (1.7, 4.9)	3.05 (1.7, 4.6)	10.3 (2.6, 19.6)	3.8 (2.3, 12.8)
PTCL	*N*	*N* = 2	*N* = 1	*N* = 1	*N* = 1	*N* = 5	*N* = 10	*N* = 15
	ORR (95% CI) (%)	50.0 (1.3, 98.7)	100.0 (2.5, 100.0)	100.0 (2.5, 100.0)	0.0 (0.0, 97.5)	60.0 (14.7, 94.7)	40.0 (12.2, 73.8)	46.7 (21.3, 73.4)
	Median DoR (95% CI) (months)	4.9 (-,-)	0.97 (-,-)	19.5 (-,-)	--	4.9 (0.0, 19.5)	7.0 (1.2, 17.3)	6.5 (2.9, 14.9)

*N* = Number of Evaluable patients; ORR = Overall Response Rate; CI = Confidence Interval, DoR = Duration of Response.

## Supplementary Materials

The following are available online at https://www.mdpi.com/2072-6694/12/8/2293/s1, Table S1: Incidence of AE (AE ≥ 5%) reported in the study (Relationship-Related).
