# Supplementary material for: Phase I/Ib Study of Tenalisib (RP6530), a Dual PI3K δ/γ Inhibitor in Patients with Relapsed/Refractory T-Cell Lymphoma
Source: Cancers (Basel). 2020 Aug 15;12(8):2293. doi: 10.3390/cancers12082293 (PMC7463651; doi:10.3390/cancers12082293)
Supplement: Supplementary file 1 [file cancers-12-02293-s001.pdf]

cancers

MDPI

Article

Phase I/Ib Study of Tenalisib (RP6530), a Dual PI3K δ/γ Inhibitor in Patients with Relapsed/Refractory T-Cell Lymphoma

Auris Huen, Bradley M. Haverkos, Jasmine Zain, Ramchandren Radhakrishnan, Mary Jo Lechowicz, Sumana Devata, Neil J. Korman, Lauren Pinter-Brown, Yasuhiro Oki, Prajak J. Barde, Ajit Nair, Kasi Viswanath Routhu, Srikant Viswanadha, Swaroop Vakkalanka and Swaminathan P Iyer

Supplementary material

Table S1. Incidence of AE (AE ≥ 5%) reported in the study (Relationship-Related).

System Organ Class	Dose Escalation (*N* = 19) *n* (%), E	Dose Expansion (*N* = 39) *n* (%), E	Total (*N* = 58) *n* (%), E
**Investigations**			
Gamma-glutamyltransferase increased	2 (10.5%), 2	5 (12.8%), 7	7 (12.1%), 9
Blood thyroid stimulating hormone increased	2 (10.5%), 2	2 (5.1%), 3	4 (6.9%), 5
Neutropenia	2 (10.5%), 2	1 (2.6%), 1	3 (5.2%), 3
Blood alkaline phosphatase increased	1 (5.3%), 1	2 (5.1%), 2	3 (5.2%), 3
Alanine aminotransferase increased	6 (31.6%), 13	12 (30.8%), 18	18 (31.0%), 31
Aspartate aminotransferase increased	6 (31.6%), 15	12 (30.8%), 20	18 (31.0%), 35
**Gastrointestinal disorders**			
Dyspepsia	1 (5.3%), 1	2 (5.1%), 2	3 (5.2%), 3
Nausea	1 (5.3%), 2	2 (5.1%), 2	3 (5.2%), 4
Vomiting	2 (10.5%), 3	1 (2.6%), 1	3 (5.2%), 4
Diarrhea	3 (15.8%), 4	9 (23.1%), 10	12 (20.7%), 14
**General disorders and administration site conditions**			
Fatigue	2 (10.5%), 2	7 (17.9%), 7	9 (15.5%), 9
Pyrexia	0	3 (7.7%), 5	3 (5.2%), 5
**Metabolism and nutrition disorders**			
Dehydration	1 (5.3%), 1	3 (7.7%), 3	4 (6.9%), 4
**Skin and subcutaneous tissue disorders**			
Pruritus	1 (5.3%), 1	3 (7.7%), 3	4 (6.9%), 4
Erythema	1 (5.3%), 1	2 (5.1%), 2	3 (5.2%), 3
**Nervous system disorders**			
Dizziness	2 (10.5%), 2	3 (7.7%), 3	5 (8.6%), 5
Headache	3 (15.8%), 4	2 (5.1%), 2	5 (8.6%), 6
**Musculoskeletal and connective tissue disorders**			
Muscle spasms	2 (10.5%), 3	3 (7.7%), 3	5 (8.6%), 6
**Blood and lymphatic system disorders**			
Anemia	1 (5.3%), 1	2 (5.1%), 2	3 (5.2%), 3

n = Number of Patients with at least one event; E = Count of Events.

© 2020 by the authors. Licensee MDPI, Basel, Switzerland. This article is an open access article distributed under the terms and conditions of the Creative Commons Attribution (CC BY) license (http://creativecommons.org/licenses/by/4.0/).
